# CRISPR/Cas9-based knockouts reveal that CpRLP1 is a negative regulator of the sex pheromone PR-IP in the *Closterium peracerosum-strigosum-littorale* complex

**DOI:** 10.1038/s41598-017-18251-8

**Published:** 2017-12-19

**Authors:** Naho Kanda, Machiko Ichikawa, Ayaka Ono, Atsushi Toyoda, Asao Fujiyama, Jun Abe, Yuki Tsuchikane, Tomoaki Nishiyama, Hiroyuki Sekimoto

**Affiliations:** 1 Graduate School of Science, Japan Women’s University, 2-8-1 Mejirodai, Bunkyo-ku, Tokyo 112-8681 Japan; 2 Faculty of Science, Japan Women’s University, 2-8-1 Mejirodai, Bunkyo-ku, Tokyo 112-8681 Japan; 3 Center for Information Biology, National Institute of Genetics, 1111 Yata, Mishima, Shizuoka, 411-8540 Japan; 4 Advanced Science Research Center, Kanazawa University, Takaramachi 13-1, Kanazawa, Ishikawa 920-0934 Japan

## Abstract

Heterothallic strains of the *Closterium peracerosum-strigosum-littorale* (*C*. *psl*.) complex have two sexes, mating-type plus (mt^+^) and mating-type minus (mt^−^). Conjugation between these two sexes is regulated by two sex pheromones, protoplast-release-inducing protein (PR-IP) and PR-IP Inducer, which are produced by mt^+^ and mt^−^ cells, respectively. PR-IP mediates the release of protoplasts from mt^−^ cells during mating. In this study, we examined the mechanism of action of *CpRLP1* (*receptor-like protein 1*), which was previously identified in a cDNA microarray analysis as one of the PR-IP-inducible genes. Using CRISPR/Cas9 technology, we generated *CpRLP1* knockout mutants in mt^−^ cells of the *C*. *psl*. complex. When the knockout mt^−^ cells were mixed with wild-type mt^+^ cells, conjugation was severely reduced. Many cells released protoplasts without pairing, suggesting a loss of synchronization between the two mating partners. Furthermore, the knockout mutants were hypersensitive to PR-IP. We conclude that CpRLP1 is a negative regulator of PR-IP that regulates the timing of protoplast release in conjugating *C*. *psl*. cells. As the first report of successful gene knockout in the class Charophyceae, this study provides a basis for research aimed at understanding the ancestral roles of genes that are indispensable for the development of land plants.

## Introduction

Sexual reproduction occurs in a wide variety of organisms, including plants, animals, and fungi. In sexual reproduction, haploid gametes of different types, which are genetically or developmentally determined, recognize each other and fuse to form a diploid zygote. In contrast to our increasing understanding of molecular mechanisms associated with sexual reproduction in higher plants^1,2^, the evolution of these processes is still uncertain. Land plants are thought to have evolved from ancestors in charophycean algae^3,4^. Charophycean algae are paraphyletic and contain six monophyletic lineages: Charales, Coleochaetales, Zygnematales, Klebsormidiales, Chlorokybales, and Mesostigmales. Until now, genomic information has only been reported in *Klebsormidium nitens* NIES-2285 (Formerly *K*. *flaccidum*)^5^, however, some transcriptomic analyses are reported using some members of charophyceans^6,7^. Recent phylogenetic analyses indicated that Zygnematales are the closest living relatives of land plants^8,9^.

To study the molecular mechanism of sexual partner recognition in charophycean algae, we focused on the *Closterium peracerosum-strigosum-littorale* complex (*C*. *psl*. complex), a member of the order Zygnematales. The *C*. *psl*. complex is one of the most widely studied unicellular charophycean algae in terms of sexual reproduction^10,11^. Furthermore, a technique for the stable transformation of the *C*. *psl*. complex has been developed^12,13^ and its genome is currently being sequenced. Heterothallic strains of the *C*. *psl*. complex have two morphologically indistinguishable sexes, mating-type plus (mt^+^) and mating-type minus (mt^−^). Sexual reproduction is readily induced when cells of the two sexes are cultured together in nitrogen-depleted medium under light. Sexual reproduction between mt^+^ and mt^−^ cells is regulated by two sex pheromones, namely protoplast-release-inducing protein (PR-IP) and PR-IP Inducer. PR-IP is produced by mt^+^ cells and induces protoplast release from mt^−^ cells. Conversely, PR-IP Inducer is produced by mt^−^ cells and induces the production of PR-IP in mt^+^ cells. As a result of the pheromonal communication, cells of opposite mating-types form a pair and release their protoplasts to form a zygote (Fig. S1). We recently proposed a possible sexual reproduction mechanism in the *C*. *psl*. complex^11,14^. However, the receptors for these sex pheromones and the mechanism of the signal transduction have not been characterized yet.

A cDNA microarray analysis revealed 88 pheromone-inducible, conjugation-related and/or sex-specific genes^15^. Among them, we focused on two genes named *CpRLK1* and *CpRLP1* because *CpRLK1* encodes a receptor-like protein kinase (RLK), whereas *CpRLP1* encodes a leucine-rich repeat (LRR) receptor-like protein (RLP). The expression of *CpRLK1* in mt^+^ cells was stimulated by the PR-IP Inducer. While the function of *CpRLK1* was characterized using knockdown mt^+^ transformants of *CpRLK1* generated by the expression of antisense RNA^13^, the role of *CpRLP1* was not shown experimentally.

Because *CpRLP1* was a sole mt^−^ specific receptor-related gene found by the cDNA microarray analysis, we initially speculated that CpRLP1 might transduce the extracellular signal of PR-IP to the intracellular compartment. However, *CpRLP1* expression was elevated in response to application of PR-IP itself^15^, suggesting that a more complex mechanism could be at work. In this study, we analyzed transformants expressing antisense *CpRLP1* RNA and knockout lines generated by the newly established CRISPR/Cas9 system to determine the physiological function of *CpRLP1*. We report that *CpRLP1* knockout mutants showed a significant loss of conjugation and were hypersensitive to PR-IP. We conclude that CpRLP1 regulates the timing of protoplast release, which is required for cell fusion, possibly through inhibiting the action of PR-IP.

## Results and Discussion

### Characterization of CpRLP1

Because the *CpRLP1* cDNA in the EST database was a partial clone (DNA Database in Japan (DDBJ) accession no. AU295384), we cloned the full-length cDNA (2,108 bp) of this gene using 5′ RACE-PCR (DDBJ accession no. LC309274). The predicted CpRLP1 protein was composed of 589 amino acid residues with a molecular mass of 60,578 Da (Fig. S2). A signal peptide directing the protein to the endoplasmic reticulum (1–32 aa) and a transmembrane domain (525–547 aa) were predicted (www.ebi.ac.uk/interpro/). The predicted extracellular domain contained 7 putative asparagine-linked glycosylation sites and 10 putative leucine-rich repeat (LRR) domains. The predicted cytoplasmic region (548–589 aa) was short and lacked a protein kinase domain.

**Figure 1:**
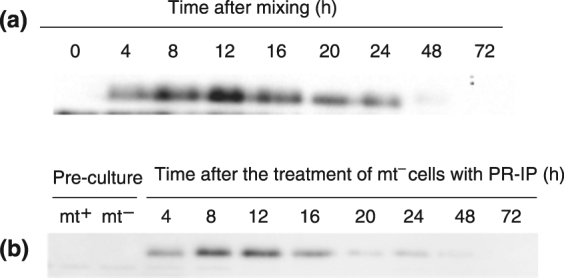
Immunological detection of CpRLP1 protein in *C*. *psl*. complex cells. (**a**) CpRLP1 expression during sexual reproduction. Cells of the mt^+^ and mt^−^ strains were co-incubated in nitrogen-depleted medium and CpRLP1 levels were monitored by immunoblot analysis at various time points after co-incubation. (**b**) Cells of the mt^−^ strain were incubated in nitrogen-depleted medium containing PR-IP. Protein was isolated at regular time intervals and subjected to SDS-PAGE followed by immunoblotting with anti-CpRLP1 antibodies.

To detect the presence of CpRLP1 in samples containing a combination of mating types, we prepared antibodies against a cocktail of two synthetic peptides (402-FGGPPRGEPYFKDD-415, 435-DTDAADGGFSEGGAG-449) based on the extracellular domain of CpRLP1. CpRLP1 was immunologically detected as a band of approximately 75 kDa; this is a little larger than the predicted size (60,758 Da), probably due to glycosylation. The proteins were detected 4 h after the mating types were mixed (Fig. 1a). The intensity of the signal increased up to 12 h after mixing and then gradually declined. CpRLP1 accumulated in mt^−^ cells that had been incubated with PR-IP (Fig. 1b). CpRLP1 protein was detected as 100-, 150-, and 250-kDa bands when SDS-PAGE was performed without reducing reagent (Fig. S3), suggesting that CpRLP1 forms heterodimers with unknown molecules or homodimers through disulfide bonds.

### Evaluation of phenotypes of knockdown transformants

To investigate the role of *CpRLP1* in cell conjugation, we constructed a knockdown vector, pSA0104*_anti-CpRLP1* (Fig. S4). Antisense *CpRLP1* cDNA was cloned under the control of the native *CpHSP70* promoter (*pCpHSP70*) and introduced in mt^−^ cells via particle bombardment. The transformed cells were selected for hygromycin resistance and six mt^−^ transformants were isolated. In a previous work, we demonstrated that the *pCpHSP70* was constitutively active and useful for driving foreign genes^16^. So, we hypothesized that the *pCpHSP70* promoter would drive expression of antisense *CpRLP1* RNA and thereby reduce CpRLP1 protein levels. When mt^−^ transformants were incubated with wild-type mt^+^ cells, the resulting sexual phenotypes varied, with some having higher rates of conjugation than others (Fig. S5). Three transformants (A1H, A4H, and A11H) had reduced levels of mating and reduced expression of *CpRLP1* compared to the control transformant, which was transformed using empty pSA0104 vector. However, we did not get a line in which the *CpRLP1* was suppressed nearly completely so that the protein was not detectable in western blotting. Thus the observed phenotype could be weaker than what will be observed with a null allele mutant.

### Isolation of CRISPR/Cas9-induced *CpRLP1* mutants

**Figure 2:**
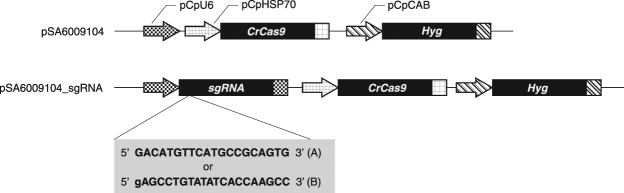
*Cas9* vector constructs with and without the *pCpU6-sgRNA* cassette. *Chlamydomonas* codon-optimized *Cas9*, *sgRNA*, and *Hyg* are under the control of the *pCpHSP70*, *pCpU6-1*, and *pCpCAB1* promoters, respectively. The plasmid backbone is pBluescript II SK^+^. An extra G at the 5′ end of the sgRNA sequence for sgRNA_B was added because the *U6* promoter requires a G for the start of transcription.

Because a null allele mutation might cause a clearer phenotype than antisense RNA technology, we used the CRISPR/*Cas9* system to knock out *CpRLP1* expression in mt^−^ cells. We introduced the pSA6009104_sgRNA_A and _B vectors (Fig. 2) into the mt^−^ cells of the *C*. *psl*. complex. Transformants were selected on media containing 50 µg/ml hygromycin. Eight clonal lines were established for the two constructs. pSA6009104, which lacks the sgRNA sequence, was introduced as a negative control.

**Figure 3:**
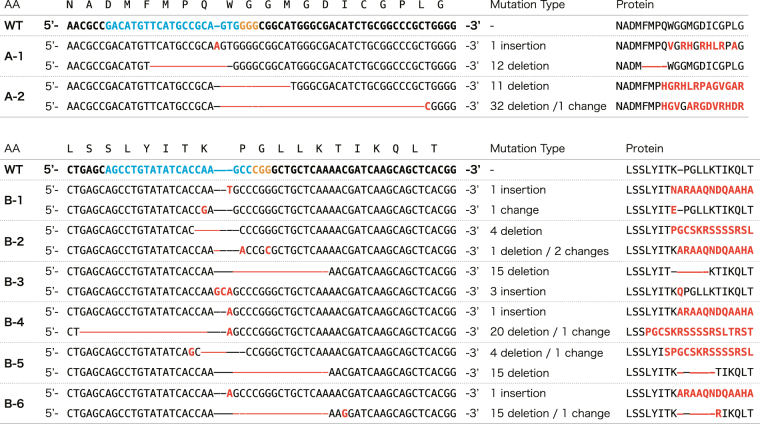
Overview of CRISPR/Cas9-induced mutations in *CpRLP1*. The wild-type (WT) sequence is shown with the protospacer adjacent motif (PAM) sequence in orange and the sgRNA target sequences (target region **A** and **B**) in blue. The corresponding amino acid (AA) sequence is depicted above the nucleotide sequence. Deletions are denoted using red dashes. Insertions and nucleotide substitutions are indicated in red letters. The amino acid sequences of the target region are listed on the right, and the altered sequences are indicated using red letters and dashes.

To confirm that the *CpRLP1* sequence was disrupted in the clonal transformants, genomic DNA spanning the target sequences was PCR amplified and sequenced. All clonal transformants showed dual sequence peaks within the target site, suggesting the presence of two copies of *CpRLP1* in the *C*. *psl*. complex NIES-68 genome. We analyzed the mutation patterns in target DNA cloned from each strain, and detected two mutation patterns in each strain (Fig. 3). Among the eight strains, three (A-2, B-2, and B-4) showed frameshift mutations in two respective gene copies and were considered to be complete *CpRLP1*-knockout mutants.

### Phenotypic characterization of *CpRLP1* mutants

**Figure 4:**
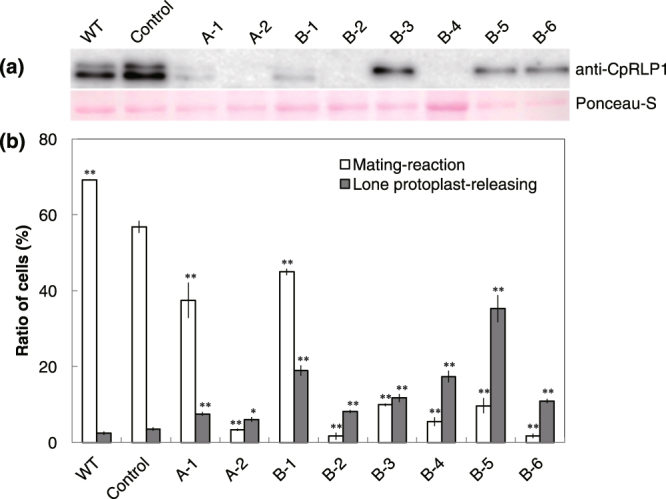
Characterization of CRISPR/Cas9-mediated *CpRLP1* knockout mutants. (**a**) Immunoblotting with anti-CpRLP1 antibodies. mt^−^ cells (wild-type, control strain, or CRISPR/Cas9-mediated *CpRLP1* mutants) were incubated with wild-type mt^+^ cells for 8 h. Protein lysates were prepared and subjected to SDS-PAGE. Ponceau-S-stained protein bands are shown as a loading control. (**b**) Status of cells 48 h after mixing. Cells exhibiting a mating reaction (pairing, protoplast-releasing from the pair, and zygote-forming cells) and lone protoplast-releasing cells are indicated by white bars and gray bars, respectively. The data represent averages of three independent experiments. Significant differences compared to the control (i.e., vector-transformed control mt^−^ cells combined with wild-type mt^+^ cells) were calculated using the generalized linear model (GLM) in R 3.2.3. and are indicated with an asterisk (*p-value < 0.01, **p value < 0.001). Vertical bars indicate SE.

The transformants were incubated with wild-type mt^+^ cells for 8 h and CpRLP1 accumulation was assayed using immunoblot assays. We detected two bands of around 75 kDa in close proximity to each other in the negative control (Fig. 4a). Both bands were absent in strains with frameshift mutations in both *CpRLP1* genes (A-2, B-2, and B-4). The bands were less prominent in strains in which only one of the *CpRLP1* genes might be functional; only the upper band was detected in B-3, B-5, and B-6, whereas both bands were detected at extremely low levels in A-1 and B-1 (Fig. 4a). Because of the presence of both bands in A-1 and B-1, in which one of the *CpRLP1* genes was disrupted, we concluded that the upper and lower bands did not correspond to the respective gene copies. Instead, the differences in protein accumulation patterns among these strains probably reflect differences in protein structure introduced by the mis-sense mutations or small deletions. Whereas strain A-1 had no change in target region B and strain B-1 had a single substitution in target region B, strains B-3, B-5, and B-6 all lacked TKPGLLK in target region B. Thus, the TKPGLLK sequence is possibly important for proper protein conformation and for the protein’s ability to function as a substrate that can be modified, resulting in faster electrophoretic mobility (e.g. dephosphorylation).

We examined the mating behaviors of transformed mt^−^ and wild-type mt^+^ cells after 48 h of co-incubation. We counted the number of mating cells (i.e., pair-forming cells, protoplast-releasing cells from the pair, and zygote-forming cells) and the number of protoplast-releasing cells without pairing (lone protoplast-releasing cells). Strains in which *CpRLP1* expression was completely absent (A-2, B-2, and B-4) and strains in which only the upper band was detected by immunoblotting (B-3, B-5, and B-6) mated less frequently than the control (vector-transformed control mt^−^ cells combined with wild-type mt^+^ cells; Fig. 4b). Significant differences were calculated using the generalized linear model (GSM) in R 3.2.3. (**p value < 0.001,^17^). In the case of the A-1 and B-1 strains, which exhibited faint lower bands in the immunoblot, mating was slightly inhibited. These data suggest that the protein represented by the lower MW band is of greater significance for the progression of mating than is the other protein. Reduced mating is consistent with the phenotype of knockdown lines that had less CpRLP1 accumulation (Fig. S5), though the degree of inhibition was smaller.

**Figure 5:**
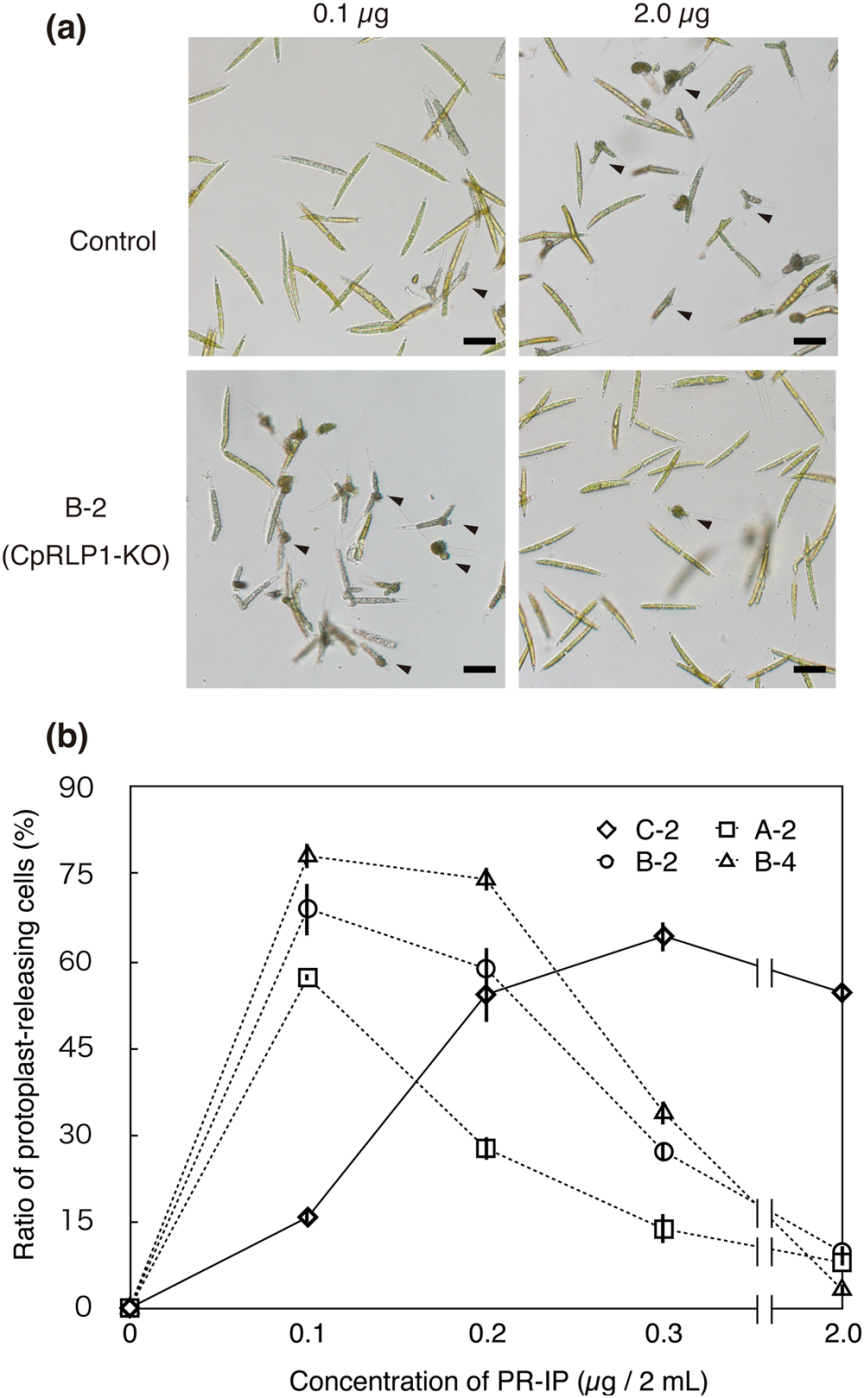
Effect of PR-IP on the induction of protoplast release. (**a**) Photographs of control and CRISPR/Cas9-mediated *CpRLP1* knockout mt^−^ cells (CpRLP1-KO) incubated with various concentrations of PR-IP. The amount of PR-IP per 2 ml of MI medium is shown above, and the names of strains are indicated to the left. Arrowheads indicate protoplast-releasing cells. Scale bar = 50 µm. (**b**) Proportion of cells releasing protoplasts in the presence of various concentrations of PR-IP. The dashed and solid lines represent data for the *CpRLP1* knockout strains (A-2, B-2, and B-4) and control strain (C-2), respectively. The data represent averages of three independent experiments. Vertical bars indicate SE.

In addition to the reduced mating reaction, all transformants showed a relatively high ratio of lone protoplast-releasing cells to total cells compared to the control (GSM, *p-value < 0.01, **p value < 0.001). It is known that mt^−^ cells but not mt^+^ cells occasionally release the lone protoplasts without pairing during sexual reproduction in the *C*. *psl*. complex^18^ and that the lone protoplast release is induced by the sex pheromone, PR-IP, which is released by mt^+^ cells^10,14^. The observation that the *CpRLP1* mutants had relatively higher rates of protoplast release without pairing than did the wild type suggests that the *CpRLP1* mutants impair the normal response to the PR-IP. The mt^−^ cells can be induced to release protoplasts in the absence of mt^+^ cells by the addition of purified PR-IP^18^. When the control transformant was exposed to various amounts of purified PR-IP, the ratio of protoplast-releasing cells to total cells increased with increasing concentrations of PR-IP up to 0.3 µg/2 ml. However, the ratio decreased with a further increase in PR-IP concentration. These results are in agreement with our previous study^18^. By contrast, the release of protoplasts by the *CpRLP1* knockout mutants peaked at 0.1 µg PR-IP/2 ml and decreased at higher concentrations of PR-IP (Fig. 5, Fig. S6).

The observation that mutants mated with wild-type mt^+^ cells could be explained as follows: the knockout mt^−^ cells are hypersensitive to PR-IP secreted from the mt^+^ cells. While some of the knockout cells released their protoplasts without pairing, most cells did not because the local concentration of PR-IP was high enough to prevent the mutant cells from releasing protoplasts.

### Conclusion and perspective

In this study, we successfully established a CRISPR/Cas9-based gene knockout system for the *C*. *psl*. complex. Using this system, we revealed the phenotypes of *CpRLP1* knockout mutants and evaluated the role of CpRLP1 in successful mating of the *C*. *psl*. complex. Knockout of *CpRLP1* markedly elevated the sensitivity of mt^−^ cells to PR-IP, suggesting that CpRLP1 acts as a negative regulator of PR-IP. With the knockdown phenotype alone appeared difficult to reach this conclusion because some activity was remaining and the phenotypes were thus weak, although the reduced mating phenotype was consistent with both experiments.

Based on results described here and previously^19,20^, we postulate that the following series of events occurs during sexual reproduction in the *C*. *psl*. complex. Under conditions that favor mating, mt^−^ cells differentiate into sexually competent cells^19^ and an unknown receptor for the 19-kDa subunit of PR-IP appears on the cell surface^20^. Once PR-IP is detected by the receptor, the mt^−^ cells start to express genes such as *CpRLP1* that facilitate the progression of sexual reproduction^15^. As a result, CpRLP1 appears on the cell surface and might form a heterodimer with the receptor (or a homodimer with itself). Following formation of the dimer, the hypersensitivity to PR-IP is reduced, which in turn, mediates protoplast release after sexual pair formation.

In general, it is considered that RLPs form receptor-complexes with RLKs and are involved in the adaptation to environmental changes and the developmental programs through the specific recognition of ligands^21–25^, however, only a few RLPs have been functionally characterized^21,26^; e.g. CLAVATA2^27,28^ and TOO MANY MOUSE (TMM)^29,30^ etc.

Recently, we identified *CpRLK2* from an EST database and found that the extracellular domain of the deduced protein shares 40% amino acid sequence similarity with that of *CpRLP1*. The position of cysteine residues was largely conserved between CpRLP1 and CpRLK2. Because this gene is expressed only in sexually differentiated mt^−^ cells, it may be a receptor for PR-IP and may form a heterodimer with CpRLP1, like as a case of ERECTA family receptor kinases and TMM^29^. We are now trying to establish *CpRLK2* knockout mutants to confirm that CpRLK2 is a receptor for PR-IP. In addition, the heterodimerization of CpRLP1 and CpRLK2 as well as the binding of PR-IP to CpRLK2 and/or CpRLP1, should be demonstrated to clarify the mechanism of PR-IP-reception and negative regulation by CpRLP1. In conclusion, this work contributes to our understanding of signaling mechanisms during the sexual reproduction of the *C*. *psl*. complex. Not restricted in the *C*. *psl*. complex, we believe that our reverse genetics approach using the CRISPR/Cas9 system can be used to characterize unknown genes in the order Zygnematales.

## Methods

### Strains and vegetative culture conditions

Strains of heterothallic *Closterium peracerosum-strugosum-littorale* complex (*C*. *psl*. complex) used in this study included NIES-67 (mt^+^) and NIES-68 (mt^−^). These were obtained from the National Institute for Environmental Studies (Ibaraki, Japan). Vegetative cells were cultured in nitrogen-supplemented medium (C medium; http://www.nies.go.jp/biology/mcc/home.htm), as previously described^18^.

### Mating culture conditions

Sexual reproduction was induced in vegetative cells of the *C*. *psl*. complex that had been cultured for two weeks (late-logarithmic phase). The cells were harvested, washed three times with nitrogen-depleted medium (MI medium;^31^), and incubated separately in 72 ml of fresh MI medium in 300 ml Erlenmeyer flasks (1.0 × 10^5^ cells ml^−1^) under continuous light (110 µmol photons m^−2^ s^−1^) for 24 h (pre-culture). Pre-cultured cells of both mating types (1.0 × 10^5^ each) were mixed in 2 ml fresh MI medium. After 48 h incubation, cells were fixed using 0.6% glutaraldehyde and the number of cells undergoing sexual reproduction were counted using an Olympus IX83 inverted microscope (www.olympus-ims.com) with a UPlanFLN4xPH, UPlanFLN10 × 2PH, or LUCPlanFLN20xPH objective lens. Images were captured using a DP80 digital camera system and cellSens Dimension software (ver. 1.9, Olympus). The experiments were repeated at least three times. Significant differences were calculated using the generalized linear model (GLM) in R 3.2.3^17^. For immunoblot analysis, pre-cultured cells of both mating types (3.0 × 10^6^ each) were mixed in 72 ml fresh MI medium in 300 ml Erlenmeyer flasks, and harvested as described^13^ at 4, 8, 12, 16, 20, 24, 48, and 72 h after the mixing.

### Cloning of *CpRLP1* cDNA and sequence analysis

Complete sequences of EST clones (4-01G01, CL27_E10, and E-41) spanning part of *CpRLP1* ^15^ were determined using the Multi-capillary Automated DNA Sequencer CEQ 2000XL (Beckman Coulter, www.meretics.com) according to the manufacturer’s instructions. cDNA synthesis from total RNA was performed using a 5′-RACE System for Rapid Amplification of cDNA Ends Kit (Invitrogen, www.lifetechnologies.com) according to the manufacturer’s instructions. The 5′ regions of genes were amplified using a gene-specific primer (4-01G01-3′) and 5′-RACE anchor primer (Invitrogen). The PCR conditions were 95 °C for 2 min; 35 cycles at 95 °C for 0.5 min, 57 °C for 45 s, and 72 °C for 5 min; and 72 °C for 5 min using Ex taq (Takara Bio, www.takara-bio.com). Nested PCR using the diluted PCR product was performed using a gene-specific primer (4-01G01-5′-race) and universal amplification primer (Invitrogen). The PCR products were separated on a 1.5% agarose gel, collected using the SUPREC 01 column (Takara), ligated into the pGEM T-easy vector (Promega, www.promega.com), and transformed into DH5α *E*. *coli* cells. Plasmid DNA was purified using the QIAprep Spin Miniprep Kit (Qiagen, www.qiagen.com/). Using sequence information from the insert DNA, full-length cDNA was amplified from primary cDNA prepared using a GeneRacer Kit with a 4-01G01-S1 primer and a GeneRacer 3′ primer. The PCR conditions were 95 °C for 2 min; 5 cycles at 95 °C for 0.5 min, 72 °C for 4 min; 5 cycles at 95 °C for 0.5 min, 70 °C for 4 min; 22 cycles at 95 °C for 0.5 min, 68 °C for 4 min; and 68 °C for 10 min. Full-length cDNA was cloned into the pGEM T-easy vector and the sequence was confirmed. The sequence was deposited in the DNA Data Bank of Japan (DDBJ) under the accession number LC309274. All primer sequences used in this study are listed in Table S1. Sequence analysis was performed using the Genetyx software package (Software Development, www.genetyx.co.jp). The deduced protein sequence was analyzed using InterPro program (http://www.ebi.ac.uk/Tools/pfa/iprscan/).

### Construction of *CpRLP1*-knockdown vectors

To construct the pSA0102_*anti-CpRLP1* vector, *CpRLP1* cDNA was PCR amplified from the cDNA clone using *CpRLP1-5*′*-BamHI* and *CpRLP1-3*′*-SpeI* primers (Table S1) and inserted between the *BamH*I and *Spe*I sites of pSA0102^13^ in the antisense direction using a Ligation Kit (Takara Bio, www.takara-bio.com) according to the manufacturer’s instructions. To construct the pSA0104_*anti-CpRLP1* vector, pSA0102_*anti-CpRLP1* was digested with *Xba*I and *Xho*I and the *CpRLP1* fragment was inserted between the *Kpn*I and *EcoR*I sites of the pSA0104 vector^13^ using a GENEART Seamless Cloning and Assembly Kit (Life Technologies, www.lifetechnologies.com) according to the manufacturer’s instructions.

All PCRs for plasmid construction were carried out using KOD-plus NEO DNA polymerase (TOYOBO, lifescience.toyobo.co.jp). Amplified PCR products were purified using a High Pure PCR Cleanup Micro Kit (Roche, roche-biochem.jp) according to the manufacturer’s instructions. Primer sequences used in this study are listed in Supplemental Table S1. The sequences of the resulting plasmids and direction of the inserts were verified using the CEQ8000 Genetic Analysis System (Beckman Coulter, www.beckmancoulter.co.jp) and the DTCS-Quick Start Kit (Beckman).

### Construction of *Cas9* vector targeting *CpRLP1*

Based on the preliminary genome database of the *C*. *psl*. complex, which is composed of raw sequence read data (DRA005947, DDBJ), we identified three putative *U6* snRNA genes. One of these genes was amplified from genomic DNA of mt^−^ cells using gene-specific primers (CpU61-S1-geneart and CpU61-A1-geneart, Table S1) and was cloned into the *BamH*I and *EcoR*I sites of the pBluescript II SK^+^ vector using a Gibson Assembly Cloning Kit (New England Biolabs, www.neb.com) according to the manufacturer’s instructions. The sequence was deposited in DDBJ under the accession number LC310798. The sequence was highly conserved among various organisms (Fig. S7).

To create pSA6009104, a codon-optimized version of *Cas9* from *Chlamydomonas reinhardtii* (*CrCas9*) was used^32^, because the codon usage of the *C*. *psl*. complex is similar to that in *C*. *reinhardtii* ^16^. *CrCas9* was PCR amplified using CrCas9-5-geneart and CrCas9-3-geneart primers and cloned into the *Xho*I site of the pSA0104 vector^13^, downstream of the *CpHSP70* gene promoter, using a GENEART Seamless Cloning and Assembly Kit. Then, the region of the *CpU6-1* promoter upstream of the endogenous *Kpn*I site was PCR amplified using CpU6-F-geneart and CpU6-R-geneart primers and cloned into the *Kpn*I site of the vector containing *CrCas9*. This vector was named pSA6009104 for CRISPR/Cas9-mediated editing of the *C*. *psl*. complex (Fig. 2).

Two sgRNA sequences for *CpRLP1* (sgRNA_A: 350-GACATGTTCATGCCGCAGTG-369, and sgRNA*_*B: 542-AGCCTGTATATCACCAAGCC-561; Fig. S1) were selected in the N-terminal region of the *CpRLP1* coding sequence using the “Guide RNA Target Design Tool” (https://wwws.blueheronbio.com/external/tools/gRNASrc.jsp). Two sgRNA expression cassettes containing a 120-bp fragment of the *CpU6-1* promoter downstream of the endogenous *Kpn*I site, one of the two target sequences for sgRNA, and the sgRNA scaffold were synthesized commercially (FASMAC, www.fasmac.co.jp) and inserted into the pSA6009104 vector. For sgRNA_B, an extra G at the 5′ end of the sgRNA sequence was added because the *U6* promoter requires a G for the start of transcription^33^.

### Transformation of the *C*. *psl*. complex

The constructs were linearized using *Not*I restriction endonuclease and the DNA was introduced into mt^−^ cells using particle bombardment as described^34^. Eight independent clonal lines were established by picking a single cell from hygromycin-resistant colonies.

### Detection of target site mutations in *CpRLP1*

Genomic DNA was extracted as described^34^. *CpRLP1* genomic DNA was PCR amplified using RLP1-S1 and RLP1-A1 primers, and cloned between the *Eco* *R*I and *Bam* *H*I sites of the pBluescript II SK^+^ vector using a Gibson Assembly Cloning Kit. The sequence was determined using RLP1-S2 and RLP1-S4 primers for target A and B, respectively.

### Preparation of anti-CpRLP1 antibody and immunoblot analysis using anti-CpRLP1 antibody

Two synthetic peptides (A: Cys-^402^FGGPPRGEPYFKDD^415^, B: Cys-^435^DTDAADGGFSEGGAG^449^) that include part of the CpRLP1 extracellular domain were used as antigens. Two rabbits were immunized with both peptides conjugated to keyhole limpet hemocyanin. Antibodies specific to the peptides were purified using affinity columns (NHS-activated Sepharose 4 Fast Flow, GE Healthcare, www.gelifedciences.com) coupled with both peptide-A and -B. The purified antibody was divided into aliquots and stored at −80 °C until needed.

Cells undergoing sexual reproduction were harvested and disrupted as described^13^. Protein content of the cell lysates was measured using the Bradford method^35^ with bovine serum albumin as the standard. The samples were subjected to SDS-PAGE using a 10% polyacrylamide gel. After electrophoresis, the proteins were transferred to a nitrocellulose membrane (Optitran BA-S 85, Whatman, www.gelifedciences.com) and probed with affinity-purified anti-CpRLP1-specific polyclonal antibody. Binding of the primary antibody was detected using a horseradish peroxidase-conjugated anti-rabbit IgG antibody (Methyl Laboratories, USA). CpRLP1 protein was detected by chemiluminescence using Versadoc (Bio-rad, USA) or the Odyssey Fc Imaging System (LI-COR, USA).

### Purification and functional assay of PR-IP

PR-IP was purified as described previously^18^. The mt^−^ cells (1.0 × 10^4^ cells) that had been cultured for 24 h were incubated in 2 ml MI medium containing various concentrations of purified PR-IP. After 48 h of incubation, cells were fixed using 0.6% glutaraldehyde and the number of cells involved in the process of papillae formation and protoplast release were counted. Cell images were obtained using an Olympus microscope system (model IX-83).

### Accession Numbers

Sequence data of *CpRLP1* cDNA and of *CpU6-1* genomic DNA can be found in the DNA Database in Japan (DDBJ) under accession numbers LC309274 and LC310798, respectively. Genomic sequence data for the NIES-68 strain can be found in the DDBJ under accession number DRA005947.

## Electronic supplementary material

Supplemental information
