# Supplementary material for: CRISPR/Cas9-based knockouts reveal that CpRLP1 is a negative regulator of the sex pheromone PR-IP in the Closterium peracerosum-strigosum-littorale complex
Source: Sci Rep. 2017 Dec 19;7:17873. doi: 10.1038/s41598-017-18251-8 (PMC5736609; doi:10.1038/s41598-017-18251-8)
Supplement: Supplementary file 1 — Supplemental information [file 41598_2017_18251_MOESM1_ESM.pdf]

CRISPR/Cas9-based knockouts reveal that CpRLP1 is a negative regulator of the sex pheromone PR-IP in the *Closterium peracerosum-strigosum-littorale* complex

Naho Kanda, Machiko Ichikawa, Ayaka Ono, Atsushi Toyoda, Asao Fujiyama, Jun Abe, Yuki Tsuchikane, Tomoaki Nishiyama, Hiroyuki Sekimoto*

Supplementary Information

Supplementary Table S1. Primers used in this study.

Primer name	Sequence (5' - 3')
4-01G01-3' [1)]	GTCAGCCGCAGCAGCCTTG
4-01G01-5'-race [1)]	AGTCGCGGATCGCCATCATCGC
4-01G01-S1 [1)]	GTGAATTCCAGATAGAACACCACAGTGGCGT
CpRLP1-3'-SpeI [2)]	TTGCCACTAGTTGTATGCCTCCAGGTCCTT
CpRLP1-5'-BamHI [2)]	GCTATGGGATCCCCTCGTTCTCTGCCCTTCGCCG
CpU6-F-geneart [3)]	GGGCGAATTGGGTACAGGAAGAAATACCCACGATGCGG
CpU6-R-geneart [3)]	CAAGTAACAAGGTACCGAGAGTTTGACA
CpU61-A1-geneart [3)]	ATAAGCTTGATATCGAATTAATGAGAGCGACTGCAGGAACAA
CpU61-S1-geneart [3)]	GCTCTAGAACTAGTGGATCAGGAAGAAATACCCACGATGCGG
CrCas9-3-geneart [3)]	GCTTCATCAAATTACTCGATTACACCTTGCGCTTCTTCTTCG
CrCas9-5-geneart [3)]	TGACTAGTGGATCCCTCGATTACAAGGACGATGATGACAA
RLP1-A1 [4)]	GTCAAGCTCAGCCGTGGTGAAT
RLP1-S1 [4)]	CCTCTCCTCGTTCTCTGCCCTT
RLP1-S2 [4)]	CCTGCCGCTCTCTTAGTATCTC
RLP1-S4 [4)]	CTGCTCGCGGCACATCATTCTC
U6P/sgRNA-F-geneart [3)]	TCAAACTCTCGGTACCTTGTTG
U6P/sgRNA-R-geneart [3)]	ACCAAGTAACAAGGTACCAAAA

1) Primers used for cloning of *CpRLP1* cDNA and sequence analysis.

2) Primers used for plasmid construction for antisense expression.

3) Primers used for plasmid construction for CRISPR/Cas9 system.

4) Primers used for detection of mutation.

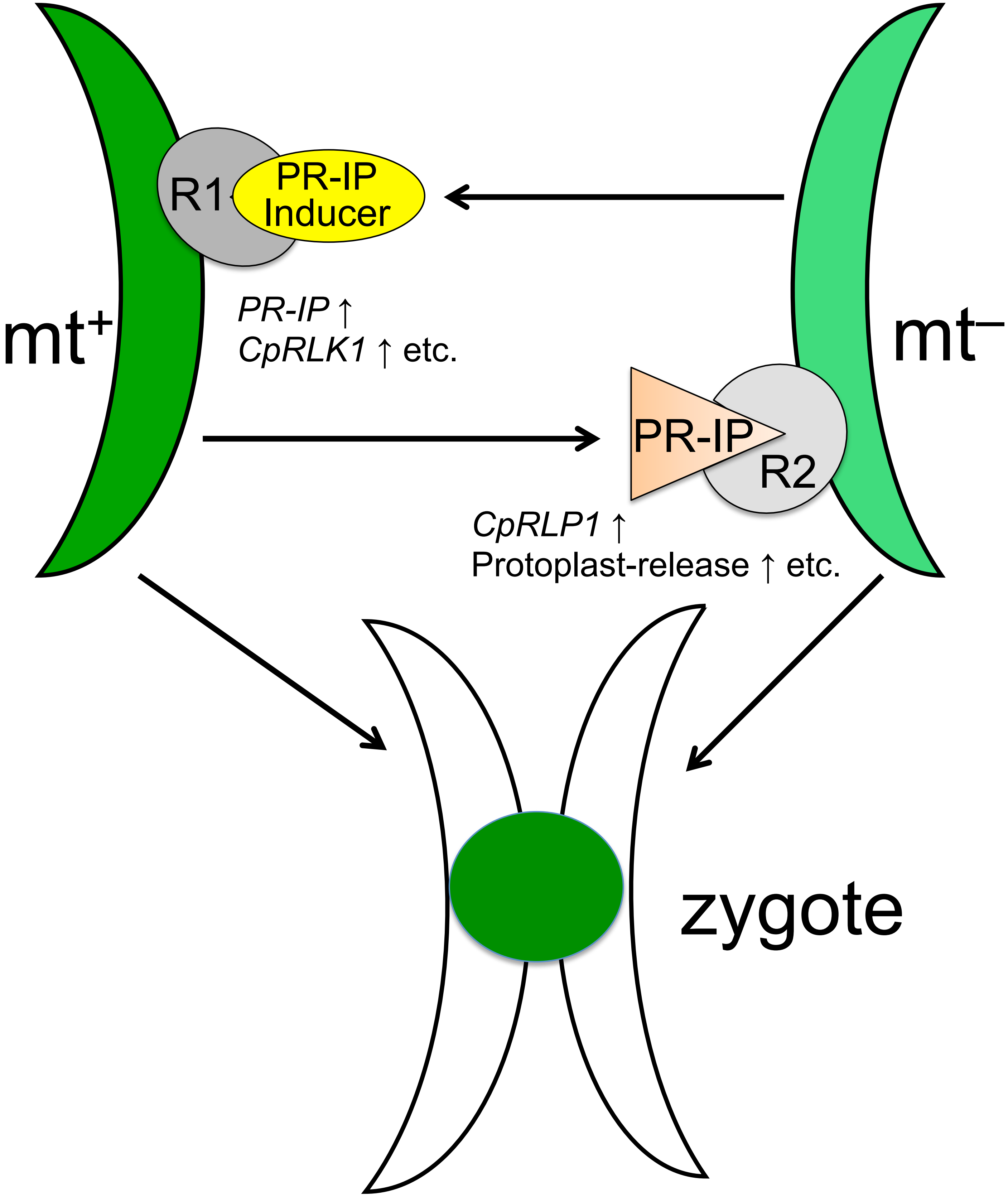

Figure S1. Schematic Illustration of the pheromonal communication during the sexual reproduction of *C. psl.* complex. PR-IP Inducer is produced by mt⁻ cells and induces the expression of some genes (e.g. *PR-IP, CpRLK1*, etc.) in mt⁺ cells. PR-IP is produced by mt⁺ cells and induces the expression of some genes (e.g. *CpRLP1* etc.) in mt⁻ cells and the protoplast release. R1; putative receptor for PR-IP Inducer on mt⁺ cell, R2; putative receptor for PR-IP on mt⁻ cell.

```
        10        20        30        40        50        60        70        80        90
CAGATAGAACACCACAGTGGCGTCTGCATCCTCTCGCTTTCTCGTTCTGTAGCGGGACTCCACTTCGCCCTCTTTCTCCGTCAGCTGCGG

       100       110       120       130       140       150       160       170       180
GAAAGCCTAAAGGCGAACCGTGCACGGCCTAGGACCGCTTTCCTTACGTCCTTTCCTTCGCTGCTGCGAGTACTCCCTGTTTTCGCTAT
                                                                                        M

       190       200       210       220       230       240       250       260       270
GGCCTCTCCTCGTTCTCTGCCCTTCGCCGCGGCCGCTGCTGCGCTGTGCGTGCTGGCCGTGGCTGTGTGCTTCGCTGTCTCCGCCGCTGC
 A  S  P  R  S  L  P  F  A  A  A  A  A  A  L  C  V  L  A  V  A  V  C  F  A  V  S  A  A  A
                                                                                 sgRNA_A
       280       290       300       310       320       330       340       350       360
TGCCGAGCCCGTCACCGGCGTCACGCCCGAGAGGGAAGTGGCGGCGATGATGGCGATCCGCGACTCGTTCGAGAACGCCGACATGTTCAT
 A  E  P  V  T  G  V  T  P  E  R  E  V  A  A  M  M  A  I  R  D  S  F  E  N  A  D  M  F  M

       370       380       390       400       410       420       430       440       450
GCCGCAGTGGGGCGGCATGGGCGACATCTGCGGCCCGCTGGGGTGGGCGCCCGAGGTGATGTGCGACATGACCGAGTCGGGCAACTACAC
 P  Q  W  G  G  M  G  D  I  C  G  P  L  G  W  A  P  E  V  M  C  D  M  T  E  S  G  N  Y  T

       460       470       480       490       500       510       520       530       540
CATCATCGGCATCTCCATCCGCGGCGCTCTGCGCGGCAGCCTCAGCCCGCTCATCCTGGACCTCGAGAACCTCAGCGCGCTGCAGCTGAG
 I  I  G  I  S  I  R  G  A  L  R  G  S  L  S  P  L  I  L  D  L  E  N  L  S  A  L  Q  L  S
 sgRNA_B
       550       560       570       580       590       600       610       620       630
CAGCCTGTATATCACCAAGCCCGGGCTGCTCAAAACGATCAAGCAGCTCACGCAGCTTAACACGCTCGACATAACCAATGTGGCGGTGGG
 S  L  Y  I  T  K  P  G  L  L  K  T  I  K  Q  L  T  Q  L  N  T  L  D  I  T  N  V  A  V  G

       640       650       660       670       680       690       700       710       720
ATTCACCACGGCTGAGCTTGACCTCTCGGGATTCACCAACCTCGAGAGCCTCACGCTGCGCAACGTGCTGATGACGGGCCCCATCGCCAA
 F  T  T  A  E  L  D  L  S  G  F  T  N  L  E  S  L  T  L  R  N  V  L  M  T  G  P  I  A  K

       730       740       750       760       770       780       790       800       810
GCTCAACCTCCCCTCGCTCCCCAACCTGCACTTCCTCGACTTGGGCCAGAACCGGCTGACGGGGGAGTTGCCGGAGGGCCTCAGCAGCAT
 L  N  L  P  S  L  P  N  L  H  F  L  D  L  G  Q  N  R  L  T  G  E  L  P  E  G  L  S  S  I

       820       830       840       850       860       870       880       890       900
CGTTCAGATGGATCTGTCCACCAACCAGCTCTCCGGCATGCTTCCCACGGACCTCCTCTCCTCCCCTAACGTCACCTCCATCATGCTGTA
 V  Q  M  D  L  S  T  N  Q  L  S  G  M  L  P  T  D  L  L  S  S  P  N  V  T  S  I  M  L  Y

       910       920       930       940       950       960       970       980       990
CAACAACAGGCTGCAGGGCGCTCTCCCGGACGTGTTCTCCTCTGCTACCAAGCTGCGCACGCTGGAGCTCTCATTGAACAAGCTCGTGGG
 N  N  R  L  Q  G  A  L  P  D  V  F  S  S  A  T  K  L  R  T  L  E  L  S  L  N  K  L  V  G

      1000      1010      1020      1030      1040      1050      1060      1070      1080
CACCCTCCCTCCTTCTCTCGCAGAGTCGCGCACTCTCACCTACATCGATCTGAGCACCAACCAGTTCGTGGGCAAGTTGCCTCGCTTCTT
 T  L  P  P  S  L  A  E  S  R  T  L  T  Y  I  D  L  S  T  N  Q  F  V  G  K  L  P  R  F  F

      1090      1100      1110      1120      1130      1140      1150      1160      1170
TGCTGCCTGCACCATCACCAACGTCTCCGCATACCTCAACGTGTCCAGCAACAAGCTCACGGGCAAGCTTCCCAACAGCCTTGCGCTCTA
 A  A  C  T  I  T  N  V  S  A  Y  L  N  V  S  S  N  K  L  T  G  K  L  P  N  S  L  A  L  Y

      1180      1190      1200      1210      1220      1230      1240      1250      1260
CACGGCCCGCAACACCGCCGCACTCACACTCGACCTGTCCTACAACGACCTCTCTGGCTCTGTCCCCGAGGGTCTCTTCGCCGCCGATGC
 T  A  R  N  T  A  A  L  T  L  D  L  S  Y  N  D  L  S  G  S  V  P  E  G  L  F  A  A  D  A

      1270      1280      1290      1300      1310      1320      1330      1340      1350
CTTCTTCTACGACCACAACGACGGGCTCTGCGGGGCGCCCCACATCCCTGCCTGCAACGCCGATGGCACGCCCTCTGCTGATAGGGCTGG
 F  F  Y  D  H  N  D  G  L  C  G  A  P  H  I  P  A  C  N  A  D  G  T  P  S  A  D  R  A  G

      1360      1370      1380      1390      1400      1410      1420      1430      1440
TGTCAATGAAACTGCTGCTGCTGGTGGCCTTTTCGGTGGTCCGCCGCGTGGGGAGCCTTACTTCAAAGATGACGGCACCATCGGGTATCT
 V  N  E  T  A  A  A  G  G  L  F  G  G  P  P  R  G  E  P  Y  F  K  D  D  G  T  I  G  Y  L

      1450      1460      1470      1480      1490      1500      1510      1520      1530
GCCTGTCGCTGATCCCGCCGACCCCGCCCCCCCTTCACCTGACACCGATGCTGCTGATGGTGGGTTTTCTGAGGGTGGTGCTGGTGAGGG
 P  V  A  D  P  A  D  P  A  P  P  S  P  D  T  D  A  A  D  G  G  F  S  E  G  G  A  G  E  G

      1540      1550      1560      1570      1580      1590      1600      1610      1620
TGGTGTTGCTGATGGTGCTGCTGACGACGGTGGTCCTTTTCCTGGTGCCGTTCCTATTTTTAACAAGGATGGCACCGTCACGTTTCAGCT
 G  V  A  D  G  A  A  D  D  G  G  P  F  P  G  A  V  P  I  F  N  K  D  G  T  V  T  F  Q  L
```

```
      1630      1640      1650      1660      1670      1680      1690      1700      1710
GCCCAATGCCTCCACCCCTGCTACCCCTTCTGCTGACACCAAGGCTGCTGCGGCTGACACTGCTGCCACTGCAGTGCCTTCCAGCACCCC
 P  N  A  S  T  P  A  T  P  S  A  D  T  K  A  A  A  A  D  T  A  A  T  A  V  P  S  S  T  P

      1720      1730      1740      1750      1760      1770      1780      1790      1800
GGCTCTCACCTGCACTTGCGAGCAGCTGTCGGCTGGCGCCATCTTCGGCATTGCCTTTGGCGCTGCTGTGCTGGCGGGTGTGCTCGTGGC
 A  L  T  C  T  C  E  Q  L  S  A  G  A  I  F  G  I  A  F  G  A  A  V  L  A  G  V  L  V  A

      1810      1820      1830      1840      1850      1860      1870      1880      1890
AGCGGTGGTGCTGACATGGCTGCACTATGGCAGCACCCGTGGGATGGCACGTGTGGAGGGCGCTGGGTTTGTGTTTGCGCCTCAGAATGG
 A  V  V  L  T  W  L  H  Y  G  S  T  R  G  M  A  R  V  E  G  A  G  F  V  F  A  P  Q  N  G

      1900      1910      1920      1930      1940      1950      1960      1970      1980
GGAGAAGATGGGCGGCGTTGGGGCCAAGGGGCCGAAGGACCTGGAGGCATACAACTAGGGGCAAGGATGAGTTTTTAAATCTCATAAGTT
 E  K  M  G  G  V  G  A  K  G  P  K  D  L  E  A  Y  N  *

      1990      2000      2010      2020      2030      2040      2050      2060      2070
TCTATGGGTTTATCGAACATGGCAGTGTTTTCTGACTTACGGATGAAGGAAAGCTATTTATCAGCGATATTTTTGGTTCTGTCATTGTTG
      2080      2090      2100      2110      2120
ATGTTTTTGTTGTGATAAATGAAGTTTTGCATTCCTGCAAAAAAAAAAAAAAAAAA
```

Figure S2. Nucleotide and deduced amino acid sequences of *CpRLP1*.
A predicted signal peptide is underlined in black. Consensus sequences for asparagine-linked glycosylation sites are underlined in orange. A predicted transmembrane domain is shown using pink letters. Peptides used to prepare specific antibodies are underlined in blue. Sequences of sgRNA targets and protospacer adjacent motif are indicated in red and blue, respectively.

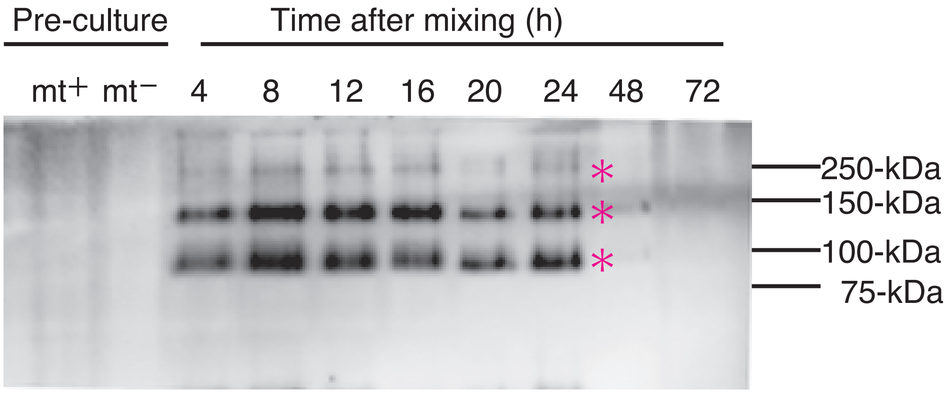

Figure S3. Effect of reducing reagent on the detection of CpRLP1 protein. Cells of mt+ and mt– strains were co-incubated in nitrogen-depleted medium and harvested at 4, 8, 12, 16, 20, 24, 48, and 72 h after mixing, as in Figure 1. Protein samples were prepared without 2-mercaptoethanol and subjected to SDS-PAGE followed by immunoblotting with anti-CpRLP1 antibodies. Protein bands are indicated with asterisks. Cropped image was displayed and original blot is shown in Figure S11.

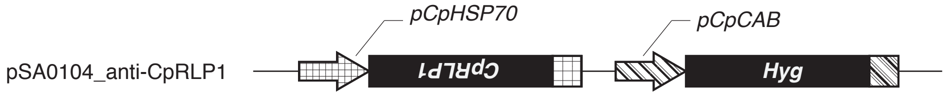

Figure S4. Schematic representation of CpRLP1 gene-silencing constructs. In pSA0104_anti-*CpRLP1*, antisense *CpRLP1* and *Hyg* were under the control of the *pCpHSP70* and *pCpCAB1* promoters, respectively. The plasmid backbone was pBluescript II SK+.

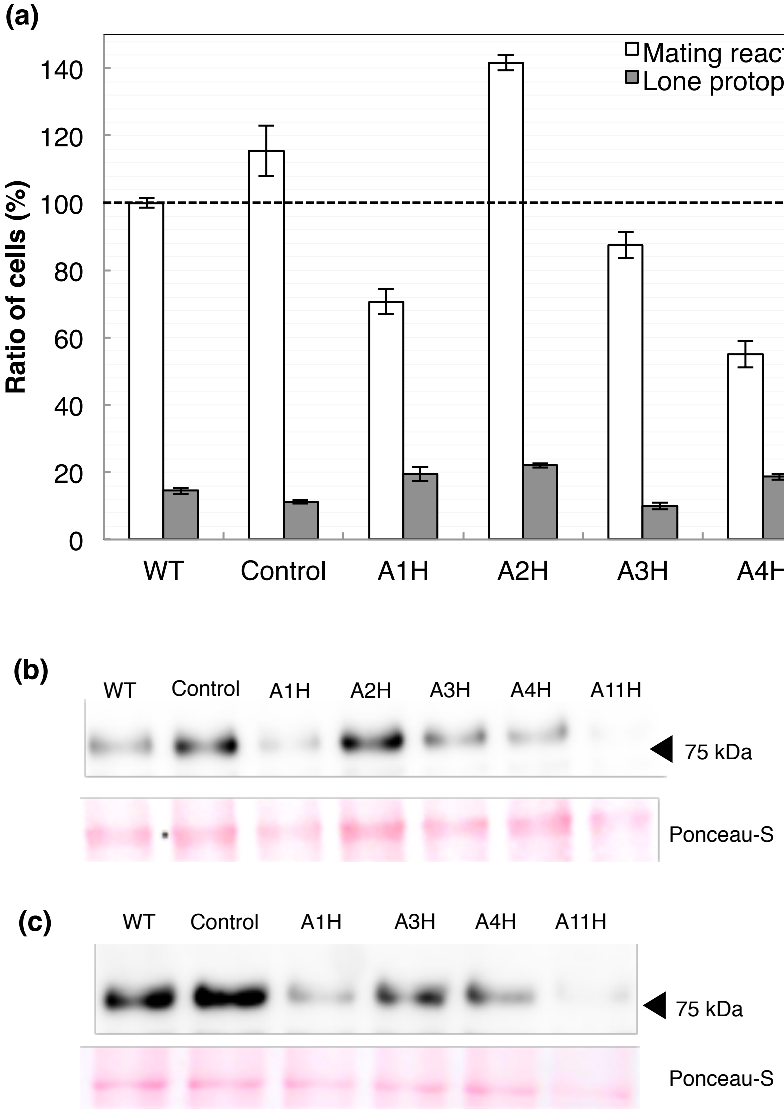

Figure S5. Phenotypic characterization of transformants expressing antisense *CpRLP1* RNA. (a) Proportion of cells exhibiting mating reactions at 48 h after co-incubation of mt^+ (wild-type) and mt^- cells (wild-type or transformants). Pairing and lone protoplast-releasing cells are indicated using white and gray bars, respectively. The ratio of cells is indicated as the relative value; the ratio of cells in wild-type (WT) is assigned to 100. Vertical bars indicate SE (n = 3). (b, c) Expression profiles of endogenous CpRLP1 protein in wild-type and transformants at 8 h after mixing as detected by immunoblotting with anti-CpRLP1 antibodies. Cropped images were displayed and original blots are shown in Figures S12 (for b) and S13 (for c).

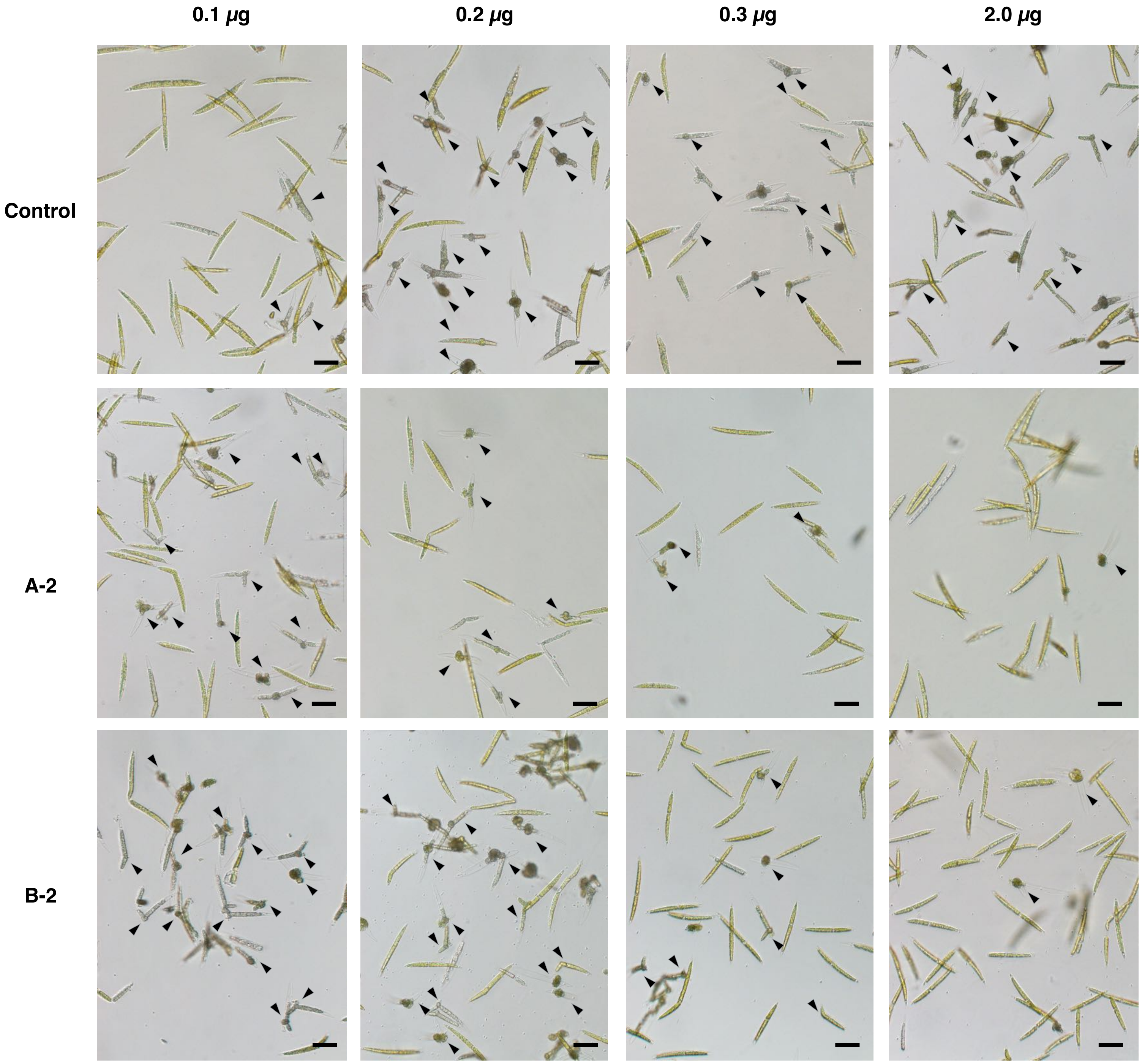

Figure S6. Effect of PR-IP on the induction of lone protoplast-releasing. Control and CRISPR/Cas9-mediated *CpRLP1*-knockout cells (A-2 and B-2) were incubated with various concentrations of PR-IP for 48 h. The amount of PR-IP per 2 mL of MI medium is shown above. Name of strains is shown on the left. Arrowheads indicate protoplast-releasing cells. Scale bar = 50 μm.

```
human_U6     1 CAGCACATATACTAAAATTGGAACGATACAGAGAAGATTAGCATGGCCCCTGCGCAAGGA
mouse_U6     1 CAGCACATATACTAAAATTGGAACGATACAGAGAAGATTAGCATGGCCCCTGCGCAAGGA
AtU6-1       1 GGGGACATCCGATAAAATTGGAACGATACAGAGAAGATTAGCATGGCCCCTGCGCAAGGA
MpU6-1       1 GGGGACATCCGATAAAATTGGAACGATACAGAGAAGATTAGCATGGCCCCTGCGCAAGGA
CpU6-1       1 CGGGACATCCGTTAAAATTGGAACGATACAGAGAAGATTAGCATGGCCCCTGCGCAAGGA

human_U6    61 TGACACGCA-AATTCGTGAAGCGTTCCATATTTTT
mouse_U6    61 TGACACGCA-AATTCGTGAAGCGTTCCATATTTTT
AtU6-1      61 TGACACGCATAAATCGAGAAATGGTCCAAATTTTT
MpU6-1      61 TGACACGCACAAATCGAGAAATGGTCCAAATTTTT
CpU6-1      61 TGACACGCACAAATCGAGAAATGGTCCAAATTTTT
```

Figure S7. Sequence alignment of *U6 small nuclear RNA* genes across various organisms and the *C. psl.* complex. Conserved sequences are shown in white font against a black background. *At*: *Arabidopsis thaliana*; *Mp*: *Marchantia polymorpha*.

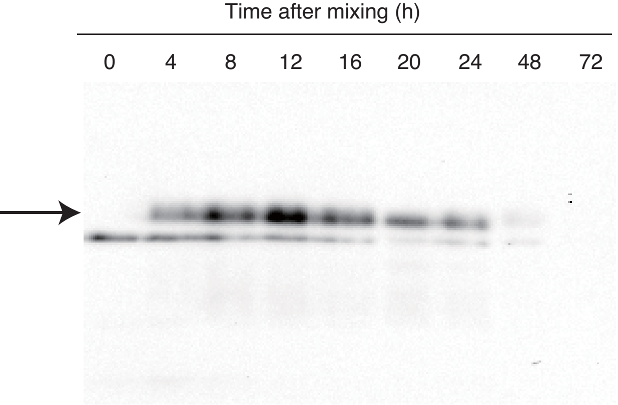

Figure S8. Original blot for Fig. 1a.

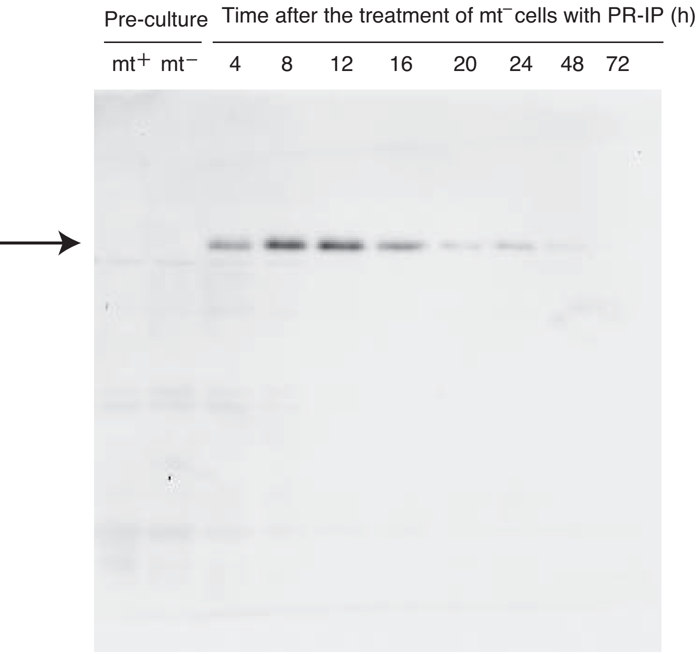

Figure S9. Original blot for Fig. 1b.

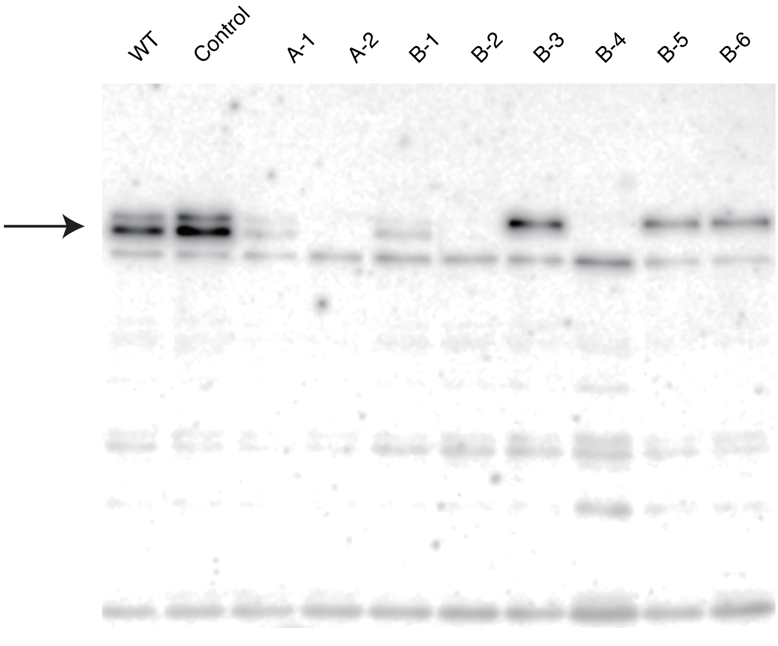

Figure S10. Original blot for Fig. 4a.

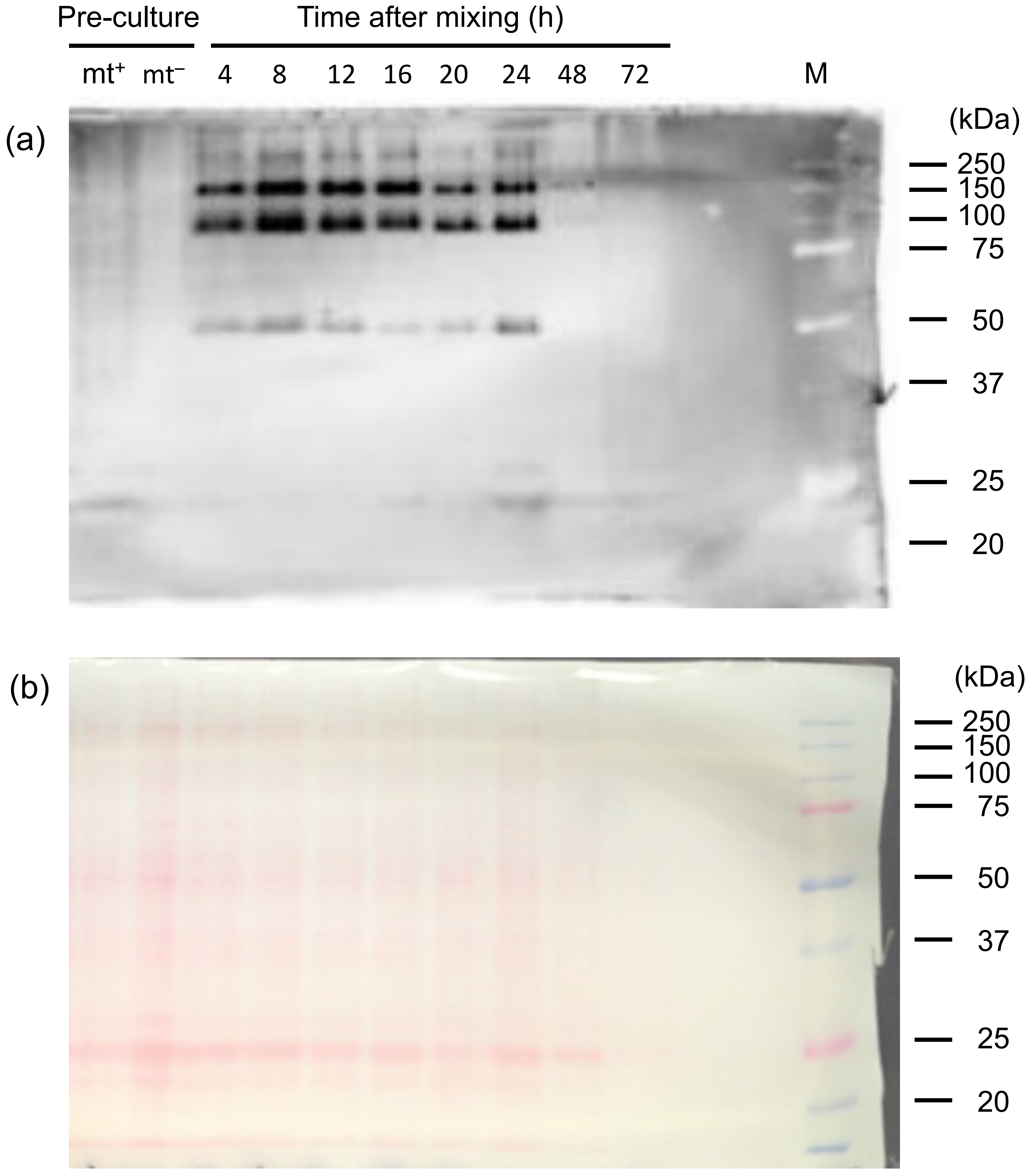

Figure S11. Original blot for Fig. S3. (a) Immunoblotting with anti-CpRLP1 antibodies. (b) Photograph of nitrocellulose membrane stained by Ponceau S. Lane M, precision plus protein dual color standards marker (BIO-RAD).

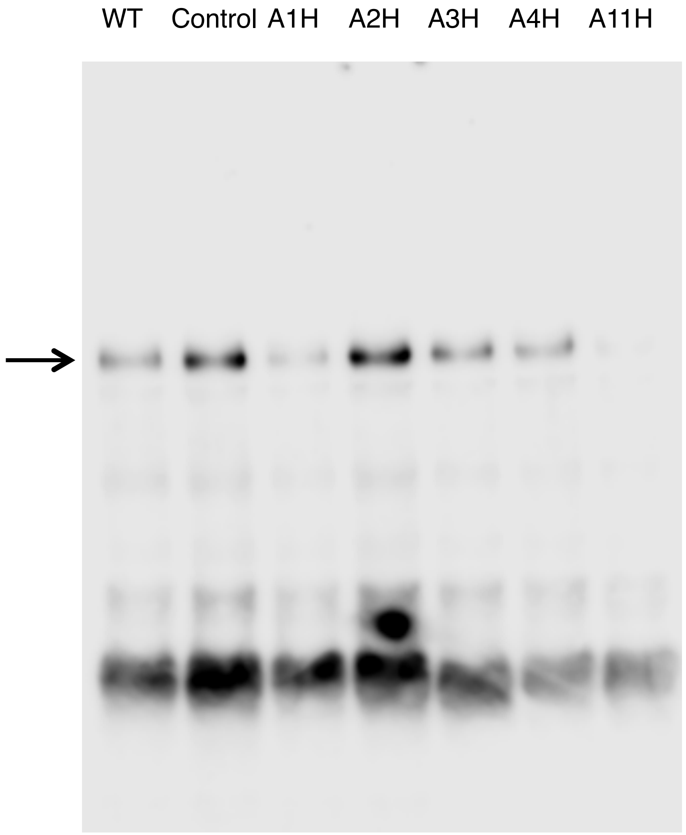

Figure S12. Original blot for Figure S5 (b).

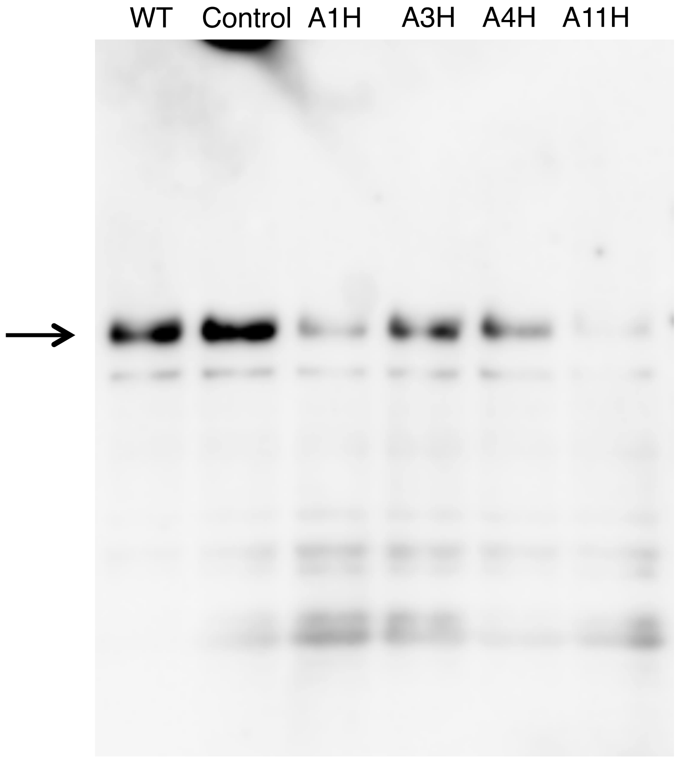

Figure S13. Original blot for Figure S5 (c).
